# Comprehensive Review of Blood Collection Techniques in Swine: Practical Guidelines for Veterinary Practice

**DOI:** 10.1155/vmi/5499360

**Published:** 2026-07-03

**Authors:** Giusy Romano, Carla Bresciani, Martina Rega, Federico Righi, Giulia Catellani, Martina Gavezzoli, Claudio Mazzoni

**Affiliations:** ^1^ Suivet Sas, Via E. Che Guevara 55, 42123, Reggio Emilia, Italy; ^2^ Department of Veterinary Science, University of Parma, Strada Del Taglio 10, 43126, Parma, Italy, unipr.it

**Keywords:** blood sampling, practitioner, swine anatomy, swine

## Abstract

Swine veterinary practice involves a range of invasive procedures, including venipuncture and catheterization, for diagnostic, therapeutic, and epidemiological purposes. With the global expansion of pig production and the growing need for effective health monitoring, particularly in the context of infectious disease control programs, such as Aujeszky’s disease and swine vesicular disease, the implementation of efficient and welfare‐conscious blood collection methods has become a priority. The selection of an appropriate venous access route is particularly critical due to the wide variation in pig size, age, and behavior, as well as the general resistance of adult pigs to human handling, which directly affects operator safety, procedural success, and animal welfare. Commonly employed sites include the jugular and ear veins, while alternative routes—such as the cranial vena cava, orbital venous sinus, tail vessels, cephalic and femoral veins, percutaneous cardiac puncture, subcutaneous abdominal vein, and the mammary vein in lactating sows—may be chosen according to the animal’s physiological status and the specific purpose of sampling. Each technique presents distinct advantages and limitations in terms of vein visibility, obtainable blood volume, repeatability, restraint requirements, and potential welfare implications. Forced restraint, required for most methods, can induce substantial stress, alter biological parameters such as serum cortisol, and compromise both sample reliability and animal well‐being. While long‐term catheterization provides repeated access with minimal handling, its use is typically confined to research settings due to the need for anesthesia, surgical expertise, and individual housing. This review presents a comprehensive anatomical and procedural overview of major blood collection techniques in pigs, critically evaluating their feasibility, invasiveness, and welfare impact. By offering a comparative analysis of each method’s suitability across clinical and field scenarios, this work aims to guide swine practitioners in selecting the most appropriate venipuncture or catheterization approach, balancing diagnostic accuracy with ethical responsibility toward animal welfare.

## 1. Introduction

Given that global pig production has increased by 140% since the 1960s, veterinary practice in swine species carries high medical responsibility [1]. Swine veterinarians play diverse roles in pig management, including clinical practice, herd health management, and food‐of‐animal‐origin quality assurance. In all these activities, swine veterinarians are recognized for their dedication to ensuring and improving animal welfare, carefully weighing the advantages and disadvantages of any procedures performed on animals.

Various stages of invasive clinical procedures must be considered [2]. In swine practice, epidemiological investigations of infectious diseases, clinical diagnoses, experimental procedures, and anesthesiology often require blood sample collection and/or vein catheterization. Circulating viruses in pig farms can affect large numbers of animals, resulting in substantial economic losses and necessitating mandatory periodic blood sampling [3, 4].

In general, evaluating seroconversion for infectious diseases requires multiple blood samples from animals of different ages to determine the most appropriate timing for vaccine administration. Monitoring herd health or performing clinical diagnoses are additional valid reasons for collecting blood samples in swine. Furthermore, proper therapeutic decisions during clinical investigations require differential diagnoses, where serological tests are essential to identify infectious agents. For example, porcine reproductive and respiratory syndrome (PRRS) requires serological monitoring and must be differentiated from other pathogens, such as *Mycoplasma hyopneumoniae* or *Brachyspira hyodysenteriae*.

Various techniques are currently used for collecting pig blood samples in different clinical settings [5]. The main venous routes reported in the literature include the following: (i) the jugular vein, (ii) the cranial vena cava, (iii) the ear veins, (iv) the tail vessels, (v) the cephalic and femoral veins, (vi) the orbital venous sinus, (vii) percutaneous cardiac puncture, and (viii) the subcutaneous abdominal vein, known as the mammary vein in lactating sows [6]. The most commonly used routes are generally the jugular and ear veins, although these approaches have limitations [7].

Swine vary greatly in size across breeds and farm types (e.g., reproduction, nursery, weaning, growing, and meat production), from small piglets to large sows and boars. The safety of veterinary personnel during procedures depends largely on animal cooperation, which typically decreases with age and may include aggressive behavior, particularly in sows during lactation [7].

Blood sampling and catheterization are invasive procedures that transiently affect animal welfare, often causing distress and potentially compromising sample quality. Forced restraint induces anxiety and discomfort in animals, indirectly influencing serum parameters, such as cortisol and other hormones. Therefore, proper selection of the venipuncture site and thorough training in rapid, safe techniques are essential to ensure both personal safety and animal welfare [8].

Adverse effects of blood sampling can be significant and may alter metabolic responses [9]. For repeated sampling, temporary catheters can be implanted under anesthesia in the jugular vein [10], auricular vein [11], mammary vein [12], or lateral saphenous vein [13], allowing sample collection with minimal animal disturbance [6]. Ear veins are often preferred for administering anesthetic drugs in experimental or surgical procedures, using butterfly needles equipped with flexible tubing [14–16].

Given the limited published literature and guidelines on this topic, this review aims to discuss blood collection techniques and provide a comprehensive veterinary perspective on venipuncture and catheterization, focusing on procedural quality and optimal animal welfare.

## 2. Materials and Methods

Relevant peer‐reviewed articles were retrieved from electronic databases, including PubMed, Scopus, and Web of Science. Search strings combined key terms related to swine venipuncture and catheterization, such as “pig blood sampling,” “swine venipuncture,” “jugular vein,” “ear vein,” “mammary vein,” “catheterization,” “animal welfare,” “stress biomarkers,” and “sampling complications.” Inclusion criteria were studies published in English that reported on swine blood collection techniques, stress indicators (e.g., cortisol, heart rate variability, behavioral measures), complications, and procedural refinements. Experimental studies, field trials, and observational reports were included, while studies lacking quantitative or comparative data, reviews without primary data, and studies on nonporcine species were excluded. All identified articles were screened by title and abstract, followed by full‐text review to extract relevant anatomical, procedural, welfare, and methodological information.

## 3. Blood Sample Collection Routes

Different blood sampling techniques are used with varying frequency in swine field practice. For clarity, this review describes techniques segmentally in a cranial‐to‐caudal direction: aural veins, orbital venous sinus, jugular vein, cephalic vein, cranial vena cava, percutaneous cardiac puncture, subcutaneous abdominal vein, mammary vein, femoral vein, and tail vessels.

After each procedure, it is advisable to apply finger pressure at the needle insertion site for a few seconds to stop bleeding. To minimize injury and bruising, no more than three attempts should be made [17]. Table 1 summarizes recommended needle sizes, blood volumes, and sampling frequency according to animal category and technique.

**TABLE 1:** Different needle sizes recommended, amount of blood, withdrawable, and repetition of sampling according to the blood sample collection route used and to the category of animals.

Blood sample collection route	Category of animal	Needle size (gauge)	Amount of blood withdrawable (mL)
Ear veins	Piglet	23	1–2
	Others	21	1–2

Orbital venous sinus	< 20 kg	Natelson tube (2.5 cm)	5–10
	20–50 kg		5–10
	> 50 kg		5–10

Jugular vein	< 50 kg	20	> 20
	> 50 kg	18	> 20

Cephalic route	< 10–15 kg	20	< 5

Cranial vena cava	< 45 kg	20	> 20
	> 45 kg	18	> 20
	Sow/boar	16	> 20

Percutaneous cardiac puncture	< 50 kg	22	10–20

Subcutaneous abdominal vein	15–50 kg	20	5–20

Mammary vein	Lactating sow	20	20

Femoral vein	< 10–15 kg	20	5–10

Tail vessels	All	20	1–2

### 3.1. The Ear Veins Route

#### 3.1.1. Anatomy

**FIGURE 1:**
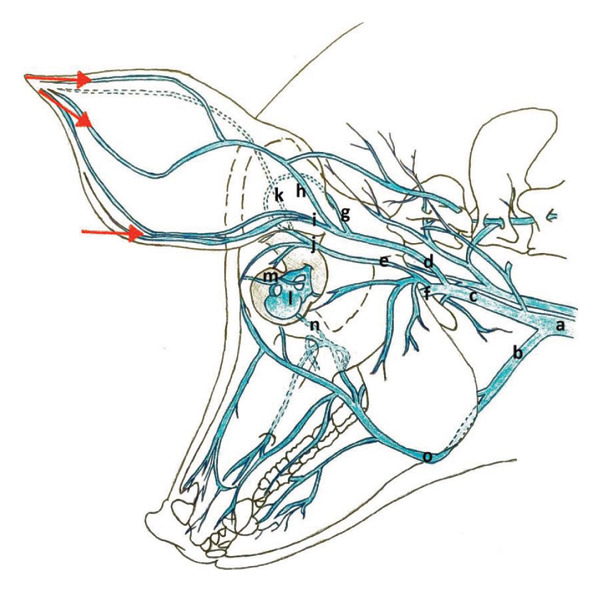
Venous vascularization of the pig’s head. (a) External jugular vein; (b) linguofacial vein; (c) retromandibular vein; (d) caudal auricular vein; (e) superficial temporal vein; (f) maxillary vein; (g) deep auricular vein; (h) intermediate auricular vein; (i) lateral auricular vein; (j) rostral auricular vein; (k) medial auricular vein; (l) ophthalmic breast; (m) dorsal external ophthalmic vein; (n) ventral external ophthalmic vein; (o) facial vein. Red arrows represent the anatomical landmarks for venous blood collection route.

The pig’s ear veins include the rostral auricular vein and transverse auricular vein, tributaries of the superficial temporal vein. The rostral auricular vein is the largest and drains into the medial auricular vein. The caudal auricular vein originates at the base of the ear, joining the lateral and middle auricular veins [18]. Prominent veins include the lateral, central, and medial veins, with the medial being the smallest (Figure 1). Pigmented ear skin can make veins harder to visualize, but because pigs do not sweat, a warm environment can dilate veins and facilitate blood collection (see Figure 1) [19].

#### 3.1.2. Restraint

The pig should be restrained using a snout rope, tightened by one operator, while a second performs the blood draw. The rope can be placed behind the canine teeth in adults, or across the lap or against the body in small pigs. Stress can be minimized by training animals to cooperate and performing the procedure in a calm environment. Local anesthetic can be applied 30 min prior to sampling [19].

#### 3.1.3. Methods

The skin should be cleaned, starting at the most distal point of the largest vein to preserve proximal sites in case of hematoma [20]. Veins can be raised using a light tourniquet or thumb pressure, or by lightly slapping the ear [20]. Blood can be collected by capillary action into a syringe or using a Vacutainer tube; capillary collection is preferred, as a constant vacuum can collapse the vein [21].

For quick tests, such as PCR, a simple prick with the needle tip may suffice to collect the required sample. No more than three attempts should be made to avoid injury or bruising.

#### 3.1.4. Adverse Effects

The ear veins are also the preferred route for anesthetic drug administration in surgical and experimental procedures using butterfly needles [14–16]. This technique is suitable for all breeds, although minipigs have small ear veins that may collapse under a strong vacuum. Possible adverse effects include bruising, hemorrhage, and infection [22].

### 3.2. The Orbital Venous Sinus Route

#### 3.2.1. Anatomy

The pig’s orbital venous system differs from that of other species, as it forms a venous sinus around the deep gland of the third eyelid instead of a venous plexus [23]. The sinus collects blood from the ventral external ophthalmic vein and the emissary vein of the round orbital hole [18].

#### 3.2.2. Restraint

Blood collection from the orbital sinus is effective if adequate restraint and experienced personnel are available [5]. Animals weighing 15–20 kg should be restrained with a snout rope behind the canine teeth [24]. Two technicians are generally needed: one to hold the animal and the other to perform sampling. The pig’s head should extend beyond the table edge, with front limbs secured rearward. The sampler should stand parallel to the animal, holding the muzzle with the nondominant hand and the syringe with the dominant hand [23].

Smaller piglets may be restrained and sampled by a single operator, using a dorsal recumbent position on a V‐shaped table. Ophthalmic anesthetic drops (e.g., proparacaine hydrochloride) can be applied a few minutes prior, although they may cause more stress than the sampling itself. General anesthesia is unnecessary, as the procedure lasts only 15–45 s [23, 25].

#### 3.2.3. Method

A Natelson tube, shortened by 2.5 cm and cut to create a triangular tip, is used to penetrate the sinus membrane. The tube is positioned at the anterior (medial) corner of the eye, inside the nictitating membrane, pointing straight out. It is tilted slightly ventrally and rearward (20°–30°) and rotated slowly to penetrate the fibrous tissue [26]. Blood flow can be enhanced by rotating the tube or adjusting the pig’s head 10°–20° toward the floor. After sampling, blood residues should be removed from the eyelids or eye area without damaging the cornea. The eye typically stops bleeding within seconds, and the pig can return to the pen immediately [23].

#### 3.2.4. Adverse Effects

Potential severe adverse effects include the following: retrobulbar hemorrhage causing hematoma, pressure, and pain; corneal ulceration due to pressure on bleeding sites or hematoma, potentially leading to keratitis, pannus, globe rupture, or microphthalmia; vision deficits or blindness from optic nerve or intraorbital damage; and eye globe penetration with vitreous humor loss [27].

### 3.3. The Jugular Vein Route

#### 3.3.1. Anatomy

**FIGURE 2:**
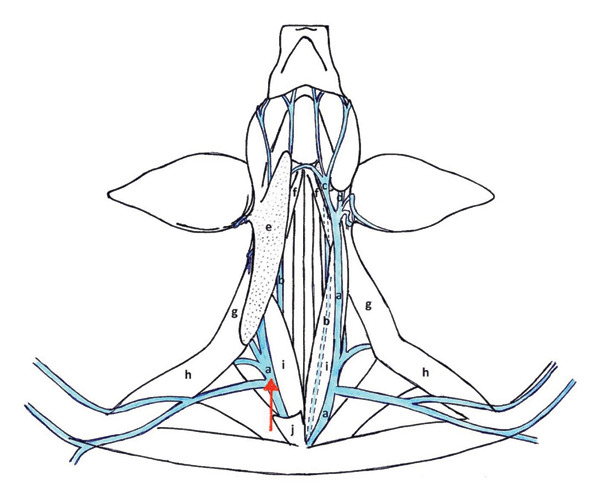
Venous vascularization of the pig’s neck. (a) External jugular vein; (b) internal jugular vein; (c) linguofacial vein; (d) retromandibular vein; (e) parotid gland; (f) homo‐ioideo muscle; (g) cleidocephalic muscle; (h) cleidobrachial muscle; (i) sternocephalic muscle; (j) neck’s sphincter muscle. Red arrows represent the anatomical landmarks for venous blood collection route.

The external jugular vein is the largest vessel in the neck of all mammals. It arises at the level of the larynx in a lateral and superficial position from the confluence of its two roots: the linguofacial and retromandibular veins. Along most of its course, it lies beneath the cutaneous muscle of the neck [18], making it easily palpable, particularly at the jugular groove, formed between the medial sternocephalic and lateral brachiocephalic muscles (Figure 2) [19]. At the base of the neck, the vein deepens, passing medially to the brachiocephalic muscle and over the ventral margin of the scalene muscles to reach the cranial thoracic opening [18].

#### 3.3.2. Restraint

This is the most commonly used blood collection route on pig farms due to its practicality across all weight categories. Restraint is necessary and varies with animal size, requiring two operators: one to restrain the animal and the other to collect the sample. Pigs under 20 kg can be held in the arms of an assistant, who supports the front legs in one hand and the head in the other. Pigs between 20 and 50 kg can be restrained on their backs in a trough or with a snout rope [19]. Animals over 50 kg require a snout rope positioned behind the canine teeth, ensuring all four feet are squarely on the ground, front legs positioned backward, and the head elevated to locate the jugular groove [6]. The right side of the neck is preferred because the right cervical nerves provide less innervation to the heart and diaphragm compared to the left vagus nerve [9].

#### 3.3.3. Method

After identifying the jugular groove (Figure 2), the needle is inserted approximately 5 cm cranial to the sternum, perpendicular to the skin, and slightly toward the midline in the deepest part of the groove. In pigs under 50 kg, the needle should be inserted more caudally and medially, closer to the sternum [19]. The needle is gently advanced with a jabbing motion until the vein is punctured and blood fills the collection tube [9, 21].

A standard disposable syringe may also be used by first breaking the vacuum seal gently, then inserting the needle at a 90° angle and aspirating to collect blood [28–30].

#### 3.3.4. Adverse Effects

Adverse effects include bruising, infection (< 1%), and hemorrhage (< 1%) [22].

### 3.4. The Cephalic Veins Route

#### 3.4.1. Anatomy

**FIGURE 3:**
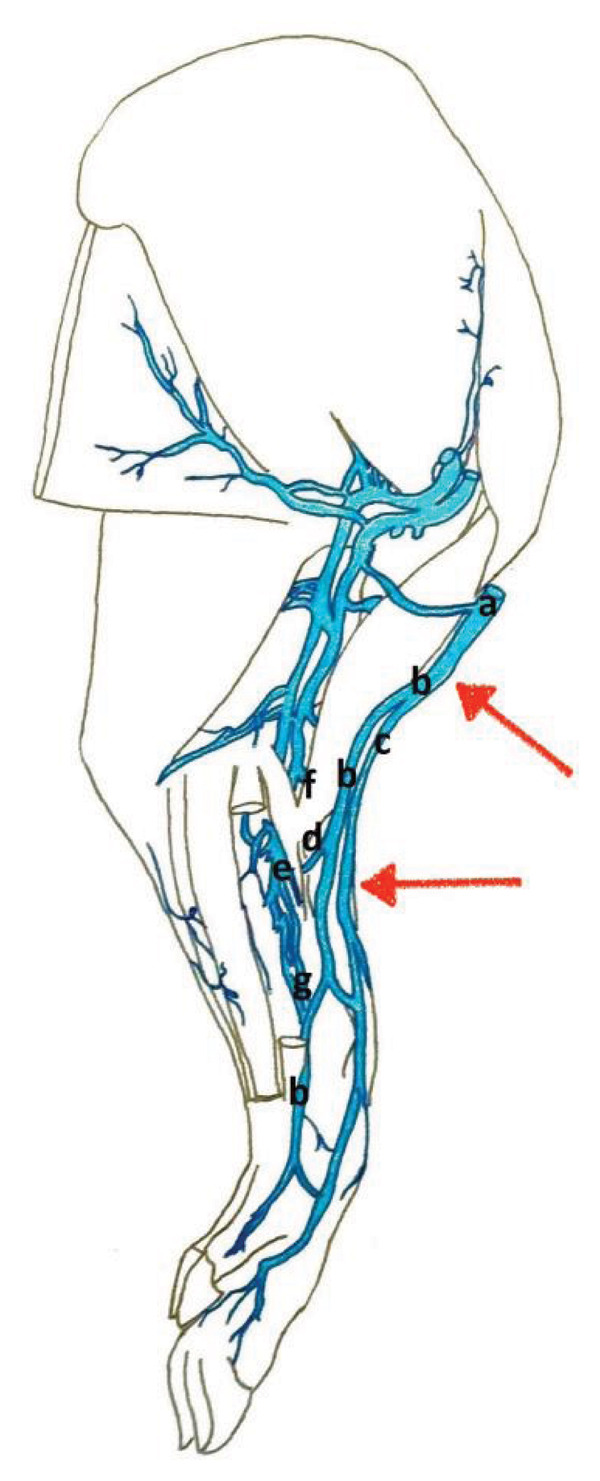
Venous vascularization of the pig’s thoracic limb. (a) External jugular vein; (b) cephalic vein; (c) accessory cephalic vein; (d) median vein of the elbow; (e) median veins; (f) proximal radial vein; (g) distal radial vein. Red arrows represent the anatomical landmarks for venous blood collection route.

The cephalic vein originates on the medial side of the metacarpal region, draining the superficial and deep palmar arches. It passes over the mid‐palmar margin of the carpus, gives rise to the distal radial vein, and then crosses the medial radius obliquely to reach the dorsal radius. Beneath the skin, it runs medially along the extensor radialis carpi muscle to the lateral elbow crease. It continues laterally to the biceps brachii, remaining superficial, and ascends ventrally between the cleidobrachialis and pectoralis muscles to the subclavian region. An accessory cephalic vein completes its territory via two roots, with the medial root forming a strong anastomosis to the distal third of the forearm (see Figure 3) [18].

#### 3.4.2. Restraint

This route is typically used in newborn or weaning piglets [19], often under sedation [13].

#### 3.4.3. Method

The piglet lies dorsally with forelimbs extended backward to expose the cephalic vein [19, 24] or with hind limbs extended to access the femoral vein [13]. The vein is visible beneath the skin and more prominent with a tourniquet or digital pressure [24]. Blood may be drawn either along the cranial surface of the radius or at the point where it joins the external jugular at the thoracic inlet. For the first method, a tourniquet is applied around the proximal radius, and the elbow is pressed to extend the limb; for the second, digital pressure is applied at the thoracic inlet alongside the trachea [13].

#### 3.4.4. Adverse Effect

This technique is limited in heavier animals due to the need to place them on their backs. Adverse effects are minimal and include bruising, infection (< 1%), and hemorrhage (< 1%) [22, 31].

### 3.5. The Cranial Vena Cava Route

#### 3.5.1. Anatomy

**FIGURE 4:**
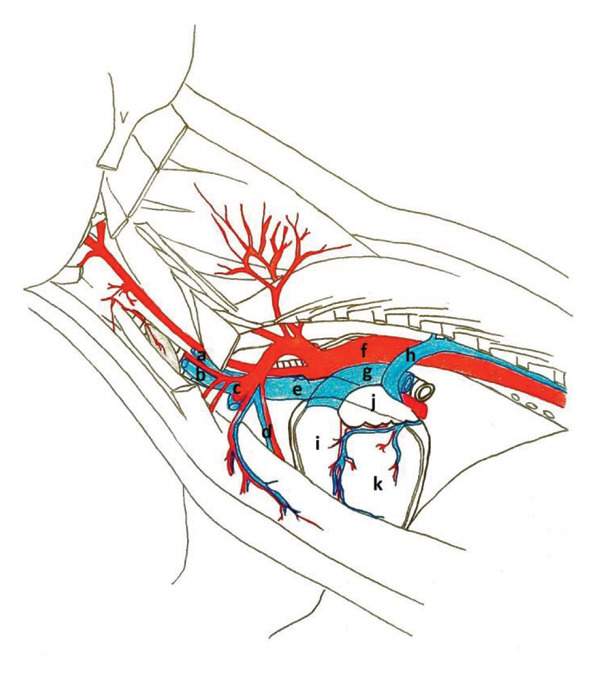
Venous and arterial vascularization of the pig’s thoracic. (a) Internal jugular vein; (b) external jugular vein; (c) axillary artery and vein; (d) internal thoracic artery and vein; (e) cranial vena cava; (f) thoracic aorta; (g) pulmonary trunk; (h) azygos vein; (i) right ventricle; (j) left atrium; (k) left ventricle.

The cranial vena cava forms from the union of the two brachiocephalic veins. It extends from the thorax to the right atrial vault in the cranial mediastinum, except for its short terminal portion within the pericardium. It runs ventrally and slightly right of the trachea, approaching the heart lateral to the brachiocephalic trunk and aorta. The right vagus nerve runs along its lateral side beneath the mediastinal pleura. Its outlet to the right atrium forms a wide, valveless orifice with only two pairs of tributaries: the internal thoracic and costocervical veins [18]. The cranial vena cava is deeper than the jugular vein, making blood collection more challenging (see Figure 4) [9].

#### 3.5.2. Restraint

Pigs over 15–20 kg are restrained using a snout rope behind the canine teeth, positioned as for jugular sampling, exposing the jugular groove [32]. Small pigs can lie dorsally with limbs pulled back and head and neck straightened, using a V‐shaped cushioned table or sling for support [21].

#### 3.5.3. Methodology

The right side is preferred to avoid the left vagus and laryngeal nerves [13]. In small pigs, the thoracic inlet is located by placing the thumb between the sternum and first rib. The needle is inserted 1 cm cranial to the sternum, slightly lateral and right of midline, angled 45° toward the opposite shoulder [13, 21, 32]. In heavier pigs, the needle is inserted at the caudal end of the jugular groove, pointing toward the top of the opposite shoulder at a 30° angle from midline and 90° from the neckline [9].

#### 3.5.4. Adverse Effect

Adverse effects include bruising, infection (< 1%), and hemorrhage (< 1%) [22].

### 3.6. The Percutaneous Cardiac Puncture

#### 3.6.1. Anatomy

The puncture site is the fourth or fifth intercostal space, medial to the apex beat, and aligned with the posterior axillary fold [33].

#### 3.6.2. Restraint

Sedation is unnecessary as the area is relatively insensitive. The pig lies supine on a trestle, nose under a restraining bar, limbs held by an assistant. One operator may sit astride the animal to stabilize the abdomen and lower limbs [33].

#### 3.6.3. Methodology

The needle is inserted at the fourth or fifth intercostal space, medial to the apex beat, perpendicular to the skin, then advanced backward and medially at a 30° angle from vertical. This approach safely reaches the left ventricle, avoiding the anterior coronary artery. Blood fills the collection tube once the ventricle is reached. Minimal movement is allowed during sampling. Animals may rest supine for ∼1 min after the procedure. Repeated punctures are possible as the procedure is typically painless [33].

#### 3.6.4. Adverse Effect

The procedure requires highly experienced personnel. Rare adverse effects include minor hematomas or pericardial effusions. Circulatory disturbances or shock are uncommon; pigs usually resume normal posture and feeding immediately, except after multiple punctures in a short period [33].

### 3.7. The Mammary Vein Route and the Subcutaneous Abdominal Vein Route

#### 3.7.1. Anatomy

The mammary vein originates primarily from the superficial epigastric veins, which connect to the cranial and caudal vena cava systems. It runs subcutaneously along the ventral wall of the thorax, adjacent to the superficial cranial epigastric veins, and drains the mammary glands. Tributaries flow in cranial and caudal directions to the superficial epigastric veins, eventually joining the deep caudal epigastric and external iliac veins [18].

During lactation, vascularization of the udder increases significantly: approximately 400 L of blood is filtered to produce 1 L of milk. The subcutaneous abdominal vein along the lateral edge of the first mammary gland becomes prominent and is referred to as the mammary vein in lactating sows [13, 18].

#### 3.7.2. Restraint

No containment or anesthesia is necessary for the mammary vein route access. The sow, housed in the farrowing crate, is naturally immobile, and a single operator can perform the blood collection [6].

The use of the subcutaneous abdominal vein route is also commonly applied in small pigs, either with manual restraint or, more commonly, under general anesthesia during experimental studies. The pig is positioned on its side with the abdomen facing the operator. Pigs over 50 kg must always be restrained with a snout rope. In all cases, two operators are required: one to restrain the animal and one to perform the blood collection [5].

#### 3.7.3. Methodology

**FIGURE 5:**
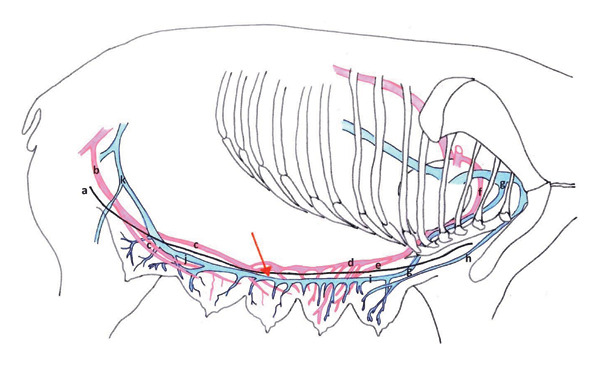
Sow’s mammary circulatory system. (a) Abdominal wall; (b) external pudendal artery; (c) branches of the pudendal artery; (d) caudal superficial epigastric artery; (e) cranial epigastric artery; (f) internal thoracic artery; (g) internal thoracic vein; (h) external thoracic vein; (i) cranial mammary vein; (j) caudal mammary vein; (k) external pudendal vein. Red arrows represent the anatomical landmarks for venous blood collection route.

Blood is collected from lactating sows (Figure 5) without repositioning the animal. The second or third mammary gland is preferred as a landmark, due to its greater venous flow. The sampler kneels laterally and inserts the needle perpendicularly into the vein, occasionally in depressions between mammary units, advancing gently until blood flows [12]. A slight camber to the hypodermic needle is often desirable. The needle is usually inserted lateral to the second mammary gland: rostrally if the right side is used and caudally if the left side is used [19, 34].

#### 3.7.4. Adverse Effect

This technique is preferred in sows with developed mammary glands, near farrowing, or during lactation. Abscess formation at the sampling site is possible if the procedure is not conducted carefully [6]. Frequent or improper use may cause thrombosis or local inflammation [34].

### 3.8. The Femoral Vein Route

#### 3.8.1. Anatomy

**FIGURE 6:**
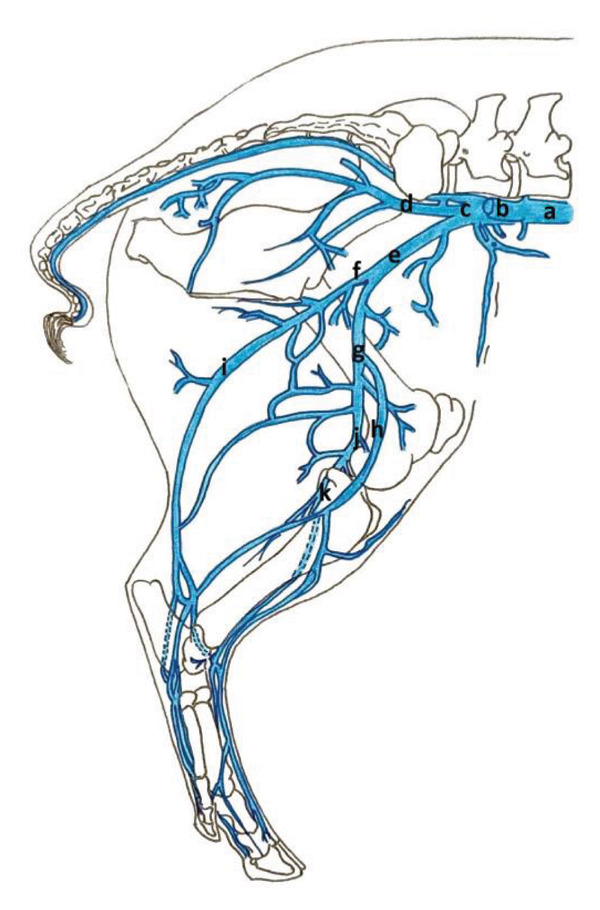
Venous vascularization of the pig’s pelvic limb. (a) Caudal vena cava; (b) right common iliac vein; (c) left common iliac vein; (d) internal iliac vein; (e) external iliac vein; (f) deep femoral vein; (g) femoral vein; (h) medial saphenous vein; (i) lateral saphenous vein; (j) popliteal vein; (k) cranial tibial vein.

The femoral vein is a major vessel of the hind limb and pelvic region. It runs beneath the distal portion of the great adductor muscle, accompanying the femoral artery through the femoral canal to the external iliac vein. It is positioned caudally and mid‐caudally to the artery and lies beneath the sartorius medially, and the gracilis and adductor muscles posteriorly. Its main tributary, the deep thigh vein, marks the boundary with the external iliac vein (see Figure 6) [18].

#### 3.8.2. Restraint

Animals are sedated prior to sampling [13].

#### 3.8.3. Methodology

Used primarily in newborn or weaning piglets [13, 19], the pig is positioned dorsally with forelimbs extended backward to access the cephalic vein or hind limbs extended to access the femoral vein [13, 24]. The femoral vein (Figure 6) lies deep under the medial edge of the gracilis muscle in the femoral groove formed by the sartorius and gracilis muscles. It can be located by palpating the pulse or following the medial saphenous artery pulse, which disappears as the vessel sinks in the groove [13].

#### 3.8.4. Adverse Effect

Improper technique can cause thrombosis, phlebitis, hematomas, subcutaneous swelling, bleeding, or infection. Stress from restraint may also affect animal welfare and sample quality [22].

### 3.9. The Tail Vessel Route

#### 3.9.1. Anatomy

**FIGURE 7:**
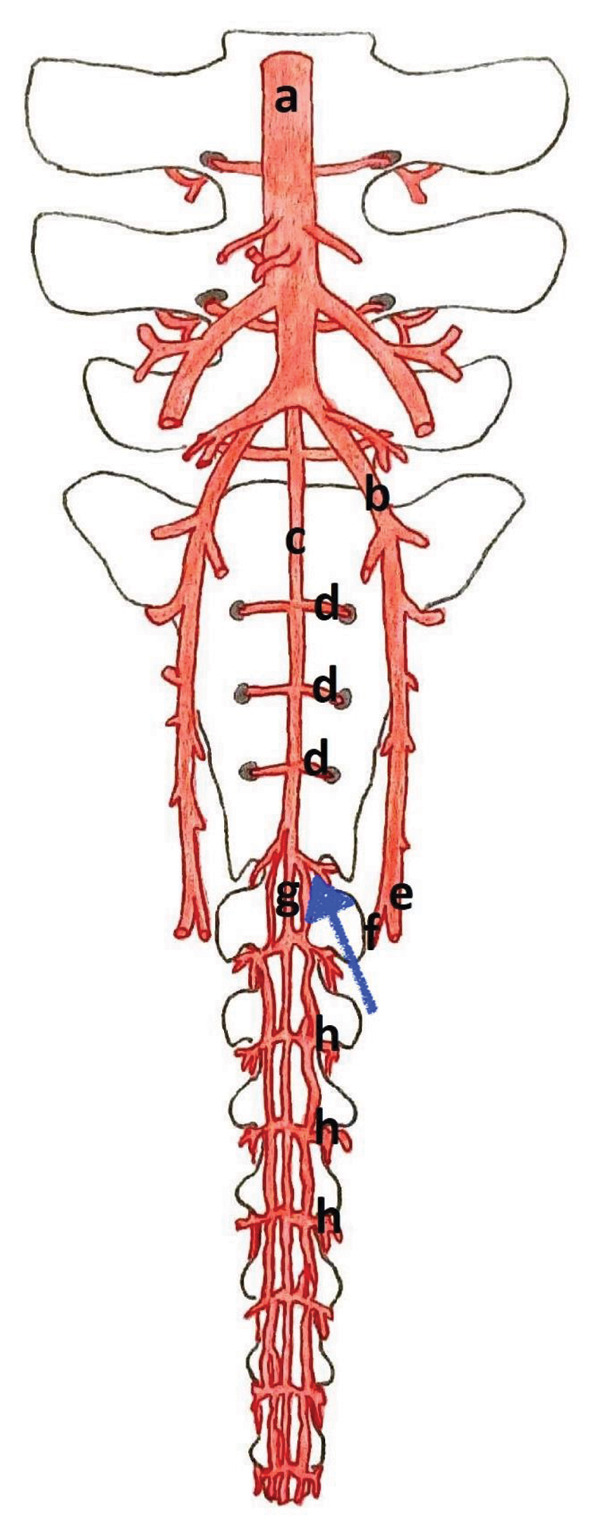
Arterial vascularization of the pig’s sacrococcygeal region. (a) Abdominal aorta; (b) internal iliac artery; (c) median sacral artery; (d) sacral branches; (e) caudal gluteal artery; (f) internal pudendal artery; (g) median coccygeal artery; (h) coccygeal branches. The course of the veins is similar.

The tail vein is a superficial ventral vessel of the tail, part of the caudal venous system. It originates at the base of the tail near the sacrococcygeal junction and runs centrally along the ventral tail surface, parallel to the median caudal artery and nerve, eventually draining into the caudal vena cava (see Figure 7) [18].

#### 3.9.2. Restraint

Animals are restrained with a snout rope [5]. In pigs > 90 kg and in sows, the tail is kept vertical; in smaller pigs, the tail is horizontal to prevent collapse of small vessels [21, 35].

#### 3.9.3. Methodology

Blood can be collected from both veins and arteries of the coccyx [5, 21]. The coccygeal veins accompany the median coccygeal artery along the ventral tail. The needle is inserted between the fourth–fifth or fifth–sixth coccygeal vertebrae. In larger pigs, the needle is angled 45° to the skin; in smaller pigs, insertion is nearly parallel [19, 35].

#### 3.9.4. Adverse Effect

Repeated use may cause thrombosis, particularly in fatty pigs. Poor technique or large‐volume sampling can lead to venous collapse [22].

## 4. Catheterization

In experimental studies requiring repeated blood sampling at fixed time points, the implantation of temporary venous catheters represents an essential technique to minimize animal disturbance while allowing accurate monitoring of physiological indicators (Scollo et al.) [6]. Among the possible access sites, the auricular vein offers a convenient route, as catheterization can be performed with the pig in an upright position without the need for sedation. After appropriate disinfection of the site, the catheter is advanced toward the base of the ear, assisted manually to facilitate passage into the larger vessels. Cinti et al. [11] reported successful maintenance of ear catheters for up to 8–9 days, permitting repeated blood sampling with minimal stress, limited to the initial restraint. Between each sampling, the catheter must be flushed with sterile heparinized saline to prevent clot formation.

The jugular vein is another commonly used site for temporary catheterization, particularly in experimental protocols that require access to a major vein. In this case, the animal is placed under general anesthesia. Following disinfection and a small skin incision, the catheter is inserted in a dorso‐caudal direction with the aid of a metal guide rod. As with auricular catheters, sterile heparinized saline must be used to flush the catheter before and after each blood collection, ensuring patency and minimizing the risk of thrombus formation [19].

Catheterization of the mammary vein is indicated primarily in lactating sows and requires general anesthesia. The sow is positioned in lateral decubitus, and the lateral thoracic region over the fold between the first and second mammary glands is shaved and disinfected. A 4‐ to 5‐cm incision is made parallel to the ventral edge of the fold to expose a venous branch of approximately 4 mm in diameter. A cannula is carefully inserted through the fascia and advanced cranially to reach the most anterior portion of the mammary vein near the xiphoid process. The cannula is then secured and sutured to the skin and placed in a protective bag affixed to the animal. Blood sampling may begin 1–2 h postsurgery, and the cannula must be flushed every 8–12 h with heparinized saline to maintain patency [12].

## 5. Discussion

Mastery of the various venous access routes and catheterization techniques is critical in swine practice, as it allows veterinarians to select the most appropriate method based on the animal’s size, age, physiological condition, and the intended purpose. Marginal ear veins, for instance, are easily visible in pigs of any size, but their small diameter limits the volume of blood that can be collected, and the vein is prone to collapse under vacuum. Therefore, ear veins are more suitable for drug infusion or anesthesia during surgical procedures than for routine blood collection in the field [5, 15, 16].

The orbital venous sinus offers an alternative route, particularly in animals weighing up to 100 kg. However, this technique is difficult to perform in field conditions, especially in animals over 15–20 kg, which require restraint with a snout rope. While placing animals in dorsal recumbency increases venous pressure and facilitates blood flow, this position is impractical for pigs standing upright. Moreover, maintaining a firm grip on the Natelson tube is essential to prevent injury in case of sudden movements [23, 25]. The requirement for anesthetic eye drops further complicates repeated sampling, making this route generally unsuitable for routine on‐field collection.

The jugular vein route remains the preferred technique for routine blood sampling in all pig categories due to its relative ease, the ability to obtain larger volumes, and suitability for repeated sampling. Proper restraint, correct positioning of the snout rope, and appropriate needle insertion techniques are critical to minimize stress, ensure successful venipuncture, and avoid injury, particularly in sows with substantial subcutaneous fat [13, 19].

Blood collection from the cranial vena cava is technically more challenging because of the vein’s deeper location. This route is less suited for repeated sampling due to the risk of hematoma or clot formation and the difficulty of applying adequate digital pressure to control bleeding [13, 32]. Percutaneous cardiac puncture allows the collection of large blood volumes and repeated sampling over minutes, hours, or days. However, it requires highly experienced personnel, strict restraint, and carries a high risk of severe complications if improperly performed, limiting its use to experimental or terminal procedures [33].

The subcutaneous abdominal, cephalic, and femoral veins are mainly used in research settings and typically require sedation or anesthesia. These routes enable repeated sampling and the implantation of temporary catheters but are impractical for routine on‐field sampling due to the time, equipment, and personnel needed [5, 13, 34]. The mammary vein, conversely, offers a practical solution for repeated sampling in lactating sows without restraint, requiring only a single operator, thus improving both human efficiency and animal welfare [6]. Tail vessels, while occasionally used in boars during semen collection, are not routinely employed due to vessel visibility issues and the need for proper restraint [5, 34].

Catheterization implants provide significant advantages for repeated sampling, minimizing handling stress, and ensuring accurate physiological monitoring. However, they require surgical skill, anesthesia, postoperative care, and protection from conspecific interference. Catheters must be regularly flushed with heparinized saline to prevent clot formation, and improper management may lead to infection, leakage, or thrombosis [5, 36]. Consequently, while invaluable for research, catheterization is not feasible for routine field sampling.

### 5.1. Adverse Effects

Adverse effects are an inherent risk of venipuncture, regardless of the technique, animal size, or context. Hematoma formation or thrombosis can occur, particularly when the needle gauge is large relative to the vein or when postsampling pressure is insufficient. Pain may be induced by excessive needle size, and infections may arise if sterile procedures are not strictly followed [28–30]. Each route carries specific risks: the ear veins may result in bruising, hemorrhage, or infection; the orbital venous sinus carries potential for corneal abrasions or more severe ocular trauma; and the jugular vein and cranial vena cava may also cause similar complications, with accidental vagus nerve puncture potentially resulting in dyspnea, cyanosis, or convulsions [13, 23, 25, 38]. Percutaneous cardiac puncture may induce hemorrhage, inflammation, or, in severe cases, sudden death due to cardiac injury [5, 33, 38]. The mammary vein is generally low‐risk, but abscess formation may occur at the sampling site if aseptic technique is not maintained [6].

### 5.2. Comparative Analysis of Blood Sampling Sites and Methods

To provide a quantitative and objective comparison, data from the selected studies were synthesized into summary tables evaluating stress responses, complication rates, blood volume yield, and reproducibility across the different venipuncture and catheterization routes. Parameters considered included serum cortisol or other stress biomarkers, hematoma formation, infection rates, ease of restraint, and required operator skill. Such comparative data allow practitioners to select the most appropriate method according to animal category, welfare impact, and procedural feasibility (see Table 2).

**TABLE 2:** Comparative assessment of major blood sampling techniques in pigs.

Sampling site/Method	Applicability (age/weight)	Stress and welfare indicators	Complication profile	Blood volume and repeatability	Practical considerations/Notes
Jugular vein	All categories (piglets ⟶ adults)	Moderate‐low stress; requires physical restraint	Bruising, hemorrhage, rare infections	High volume (10–20 mL), high repeatability	Field routine method of choice; requires 2 operators for adults
Ear veins	All categories	Relatively low stress	Bruising, hemorrhage, potential vein collapse	Low volume (1–5 mL), medium repeatability	Excellent for infusion/anesthesia; vein may collapse if excessive sampling
Mammary vein	Lactating sows	Minimal stress, often without restraint	Possible abscess formation if asepsis is not ensured	Moderate volume (5–10 mL), high repeatability	Single operator; improved welfare; limited to sows with developed mammary glands
Cranial vena cava	Adults	Moderate	Bruising, hematoma risk, possible nerve injury	High volume (10–15 mL), low repeatability	Deep vessel; difficult technique; not suitable for repeated sampling
Orbital venous sinus	Pigs < 100 kg	High potential stress (invasive, abnormal positioning)	Retrobulbar hemorrhage, corneal ulcers, potential blindness	Moderate, but not recommended	High‐risk technique; unsuitable for field use
Percutaneous cardiac puncture	Experimental use mainly	Variable stress; requires sedation/immobilization	Hemorrhage, pericardial effusion, severe trauma, potential fatality	Large volume (> 20 mL), high potential repeatability	Only for research/terminal sampling; requires high technical expertise
Subcutaneous abdominal vein	15–40 kg (experimental)	Stress reduced under anesthesia	Thrombosis or inflammation if used improperly	Moderate‐high volume; possible repeated sampling	Requires anesthesia and time; not routine in the field
Cephalic and femoral veins	Piglets/experimental	Generally low stress (sedated)	Bruising, rare infection (< 1%), hemorrhage	Variable volume; good repeatability in experimental settings	Not field‐practical without sedation
Tail vessels	Adults and young pigs	Variable stress; requires restraint	Thrombosis if repeated, venous collapse possible	Low‐moderate	Difficult visibility in the field; occasionally used in boars

## 6. Conclusions

Multiple venous access routes are available for blood sampling in swine, and the choice should be guided by the animal’s size, physiological condition, and the sampling objective. The jugular vein remains the gold standard for routine field sampling due to its accessibility, suitability across weight categories, and capacity for repeated collections, although it requires two operators and proper restraint. The mammary vein represents an innovative alternative in lactating sows, enabling efficient blood collection by a single operator without restraint, enhancing both animal welfare and labor efficiency. Tail and ear veins may be used for boars during semen collection, with ear veins also serving as preferred routes for anesthesia administration. Subcutaneous abdominal, cephalic, and femoral veins, as well as catheterization routes, are more appropriate for research purposes, allowing repeated or large‐volume sampling under sedation or anesthesia. Routes such as the orbital venous sinus, cranial vena cava, and percutaneous cardiac puncture are technically demanding and are generally unsuitable for routine on‐field use, although they may serve specific experimental or terminal applications. Overall, the selection of the appropriate venous access route requires careful consideration of the balance among practicality, sample quality, animal welfare, and operator safety.

## Funding

This research did not receive any specific grant from funding agencies in the public, commercial, or not‐for‐profit sectors. Open access publishing facilitated by Universita degli Studi di Parma, as part of the Wiley ‐ CRUI‐CARE agreement.

## Conflicts of Interest

The authors declare no conflicts of interest.

## Data Availability

Data sharing is not applicable to this article, as no new data were generated, or the article describes entirely theoretical research.

## References

[1] Kim S. W. , Gormley A. , Jang Ki B. , and Duarte M. E. , Invited Review-Current Status of Global Pig Production: An Overview and Research Trends, Animal Bioscience. (2024) 37, no. 4, 719–729, 10.5713/ab.23.0367. 37946421 PMC11016693

[2] Law J. , Swine Veterinarians—Key Players in the Pork Production Chain, Canadian Veterinary Journal. (2021) 62, no. 5, 515–516, https://pmc.ncbi.nlm.nih.gov/articles/PMC8048199/. PMC8048199 33967293

[3] Ministerial Decree, 2010, https://www.gazzettaufficiale.it/eli/id/2011/02/12/11A01746/sg.

[4] Ministerial Order, 2008, https://www.gazzettaufficiale.it/eli/id/2008/06/26/08A04434/sg.

[5] Hu C. , Cheang A. , Retnam L. , and Yap E. H. , A Simple Technique for Blood Collection in the Pig, Laboratory Animals. (1993) 27, no. 4, 364–367, 10.1258/002367793780745606. 8277710

[6] Scollo A. , Bresciani C. , Romano G. et al., A Novel Blood-Sampling Technique in Lactating Sows: The Mammary Vein Route, Veterinary Journal. (2019) 254, 10.1016/j.tvjl.2019.105397. 31836171

[7] Elane G. L. , Bauck A. G. , Hobbs K. J. et al., Review of Venipuncture and Intravenous Catheterization Techniques in Pigs, Journal of the American Veterinary Medical Association. (2024) 262, no. 10, 1–9, 10.2460/javma.24.03.0169. PMC13055995 38906168

[8] Zanella A. J. and Mendl M. T. , A Fast and Simple Technique for Jugular Catheterization in Adult Sows, Laboratory Animals. (1992) 26, no. 3, 211–213, 10.1258/002367792780740611. 1501436

[9] Zimmerman J. J. , Karriker L. A. , Ramirez A. , Schwartz K. J. , Stevenson G. W. , and Zhang J. , Diseases of Swine, Diseases of Swine, March. (2019) 1, 1108, 10.1002/9781119350927.

[10] Stukelj M. , Mihelcic D. , Butinar J. , Nemec A. , and Pecar J. , Surgical Intravenous Catheterization of Pig, Slovenian Veterinary Research. (2005) 43, 43–48.

[11] Cinti E. , Mazzoni C. , Borri E. , and De Rensis F. , Cateterizzazione Non Invasiva Della Vena Auricolare Per Prelievi Frequenti Nella Specie Suina, Large Animal Review. (2014) 20, 65–68.

[12] Trottier N. L. , Shipley C. F. , and Easter R. A. , A Technique for the Venous Cannulation of the Mammary Gland in the Lactating Sow, Journal of Animal Science. (1995) 73, no. 5, 1390–1395, 10.2527/1995.7351390X. 7665368

[13] Swindle M. M. , Smith A. C. , Laber-Laird K. , and Dungan L. , Swine in the Laboratory: Surgery, Anesthesia, Imaging, and Experimental Techniques, 2007, 2nd edition, CRC Press.

[14] Skarlandtová H. , Bičíková M. , Neužil P. et al., Might Cardiac Catheterization Influence Diurnal Rhythm of the Steroid Stress Hormones Secretion?, Physiological Research. (2012) 61, no. 1, 25–34, 10.33549/physiolres.932208. 22188113

[15] Scollo A. , Martelli P. , Borri E. , and Mazzoni C. , Pig Surgery: Cryptorchidectomy Using an Inguinal Approach, Veterinary Record. (2016) 178, no. 24, 10.1136/VR.103592. 27053253

[16] Anderson D. E. and Mulon P. Y. , Anesthesia and Surgical Procedures in Swine, 2019, Diseases of Swine, March, 10.1002/9781119350927.CH11.

[17] Marchant-Forde J. N. and Herskin M. S. , Pigs as Laboratory Animals, Advances in Pig Welfare. (2018) 445–475, 10.1016/B978-0-08-101012-9.00015-0.

[18] Barone R. , Anatomia Comparata Dei Mammiferi Domestici, Angiologia. Parte Prima: Cuore E Arterie.” Edited by Ruggero Bortolami and Emilio Callegari. (2007) 5, 15–23.

[19] Framstad T. , Sjaastad Ø. , and Aass R. A. , Bleeding and Intravenous Techniques in Pigs, 2000, The Norwegian School of Veterinary Science.

[20] Ramirez A. and Karriker L. A. , Herd Evaluation, 2019, Diseases of Swine, March, 10.1002/9781119350927.ch1.

[21] Muirhead M. R. , Blood Sampling in Pigs, Practice. (1981) 3, no. 5, 16–20, 10.1136/inpract.3.5.16. 7346487

[22] Blood Sampling: Pig|NC3Rs, 2025, https://nc3rs.org.uk/3rs-resources/blood-sampling/blood-sampling-pig#cranial-vena-cava.

[23] Dove C. R. and Alworth L. C. , Blood Collection From the Orbital Sinus of Swine, Lab Animal. (2015) 44, no. 10, 383–384, 10.1038/laban.869. 26398611

[24] Straw B. E. , Zimmerman J. J. , D’Allaire S. , and Taylor D. J. , Diseases of Swine, 9th Edition 1st Section, 2006, Blackwell Pub, 12–14.

[25] Huhn R. G. , Osweiler G. D. , and Switzer W. P. , Application of the Orbital Sinus Bleeding Technique to Swine, Laboratory Animal Care. (1969) 19, no. 8, 403–405. 4240473

[26] Diehl K. H. , Hull R. , Morton D. et al., A Good Practice Guide to the Administration of Substances and Removal of Blood, Including Routes and Volumes, Journal of Applied Toxicology. (2001) 21, no. 1, 15–23, 10.1002/JAT.727. 11180276

[27] Holtgrew-Bohling K. , Large Animal Clinical Procedures for Veterinary Technicians, 2012, 2nd edition, Elsevier Mosby.

[28] McCurnin D. M. and Bassert J. M. , Clinical Textbook for Veterinary Technicians, 2002, Elsevier Mosby.

[29] Bollen P. J. A. , Hansen A. K. , and Olsen Alstrup A. K. , The Laboratory Swine, 2010, Laboratory Swine, 10.1201/9781439815304.

[30] Sankari S. , A Practical Method of Taking Blood Samples From the Pig, Acta Veterinaria Scandinavica. (1983) 24, no. 1, 133–134, 10.1186/BF03546765. 6869143 PMC8291235

[31] Framstad T. , Sjaastad Ø. , and Aass R. A. , Blodprøvetaking På Gris, Norsk Veterinærtidsskrift. (1988) 100, no. 4, 265–272.

[32] Snook C. S. , Use of the Subcutaneous Abdominal Vein for Blood Sampling and Intravenous Catheterization in Potbellied Pigs, Journal of the American Veterinary Medical Association. (2001) 219, no. 6, 809–810, 10.2460/javma.2001.219.809. 11561659

[33] Getty R. and Ghoshal N. G. , Applied Anatomy of the Sacrococcygeal region of the Pig as Related to Tail-Bleeding, Veterinary Medicine, Small Animal Clinician. (1967) 62, no. 4, 361–367. 5182699

[34] Calvert G. D. , Scott P. J. , and Sharpe D. N. , Percutaneous Cardiac Puncture in Domestic Pigs, Australian Veterinary Journal. (1977) 53, no. 7, 337–339, 10.1111/J.1751-0813.1977.TB00244.X. 921641

[35] Broes A. , Caya I. , and Bélanger M. , Practice Tip: New Blood Collection Technique for Porcine Reproductive and Respiratory Syndrome Virus Monitoring in Boars, Journal of Swine Health and Production. (2007) 15, no. 1, 42–44, http://www.aasv.org/shap.html, 10.54846/jshap/492.

[36] Jackson I. M. D. , Cook D. B. , and Gill G. , Simultaneous Intravenous Infusion and Arterial Blood Sampling in Piglets, Laboratory Animal Science. (1972) 22, no. 4, 552–555. 4340301

[37] Dewey C. W. and Barbara Straw E. , Diseases of Swine, 2006, 9th edition, Blackwell Publishing.

[38] Friend D. W. and Brown R. G. , Blood Sampling From Suckling Piglets, Canadian Journal of Animal Science. (1971) 51, no. 2, 547–549, 10.4141/CJAS71-074.

